# NHS staff awareness, attitudes and actions towards the change in organ donation law in England—results of the #options survey 2020

**DOI:** 10.1186/s13690-023-01099-y

**Published:** 2023-05-10

**Authors:** Dorothy Coe, Natasha Newell, Mark Jones, Matthew Robb, Natalie Clark, David Reaich, Caroline Wroe

**Affiliations:** 1 Newcastle-upon-Tyne Hospitals NHS Foundation Trust, Newcastle upon Tyne, UK; 2 Newcastle University, Newcastle upon Tyne, UK; 3 NHS Blood and Transplant, Bristol, UK; 4 South Tees Hospitals NHS Foundation Trust, Middlesbrough, UK

**Keywords:** Organ donation, Legislation, Education, National Health Service

## Abstract

**Background:**

In Spring 2020 there was a change in organ donation legislation in England (UK). Much is known about public opinions to organ donation and the change in legislation, however, there is little evidence about the opinions of the NHS workforce. This study set out to understand the levels of awareness, support and action of NHS staff to this change and explore the impact of respondent demographics, place and type of work on awareness, support and action.

**Methods:**

An online survey was offered to all NHS organisations in North Thames and the North East and North Cumbria through the NIHR Clinical Research Network between July and December 2020. Participating organisations were provided with an information package and promoted the survey via email and internal staff communications. Associations were compared univariately using chi-square tests and logistic regression was used for multivariable analysis to compare findings with NHS Blood and Transplant public Kantar survey data.

**Results:**

A total of 5789 staff participated in the survey. They were more aware, more supportive, more likely to have discussed their organ donation choices with family and more likely to be on the organ donor register than the public. This increased awareness and support was found across minority ethnic and religious groups. Those working in a transplanting centre were most aware and supportive and those working in the ambulance service were most likely to ‘opt-in’ following the change in legislation.

**Conclusions:**

NHS staff in England were well informed about the change in organ donation legislation and levels of support were high. NHS staff were six times more likely than the public to have a conversation with their family about their organ donation choices. The size and ethnic diversity of the NHS workforce offers an opportunity to enable and support NHS staff to be advocates for organ donation and raise awareness of the change in legislation amongst their communities.

**Supplementary Information:**

The online version contains supplementary material available at 10.1186/s13690-023-01099-y.

## Background

In the United Kingdom there is a chronic shortage of organs for transplantation. At the end of February 2020 there were 6138 patients waiting for a transplant, with individuals dying while waiting for an organ [1]. The overall consent rate for transplant from eligible donors during the year 1 April 2019 to 31st March 2020 was 68%. Consent rates rose to 91% where a person was known to have registered an opt-in decision on the organ donation register (ODR) or made their wishes known [2]. There are known factors that affect consent rates. They are higher after brain death than circulatory death [3] and vary by geography from 61% in London to 46% in Scotland [1] and are lower in ethnic minorities (42%) than White eligible donors (72%) [2]. Consent rates are also influenced by the knowledge and skills of healthcare professionals [4], in particular their ability to sensitively communicate donation procedures to enable full understanding.

Efforts have previously been made by the National Health Service Blood and Transplant (NHSBT) to increase consent rates [5], however these fell short of the 2020 target for a 80% consent rate. To further improve organ donation rates England has followed a worldwide trend to move to an opt-out consent for organ donation [6]. This follows a similar move by Wales in 2015. In England the narrative around the change to opt-out began in 2017 when the then prime minister vowed to change the law. In February 2019 the organ donation law was passed through parliament and in March 2019 it received Royal Assent, with the law coming into effect in May 2020 [7]. Under the law all adults in England are considered to have agreed to donate their organs when they die, unless they record a decision not to donate (opt-out) or are in one of the excluded groups [8]. Alongside the legislative change, England continues to operate an opt-in system where individuals can actively opt into the organ donation register. To coincide with the increasing narrative around the law change NHSBT launched a public awareness campaign in April 2019.

International expert opinion considers opt-out as only one of several factors that impact on organ donation rates [6, 8, 9]. Law change needs to be accompanied by improved awareness and education to maximise opportunities for transplant. Additional areas that are known to impact on donation rates include the standardisation of donor screening, the training given around approaching relatives and the usefulness of public awareness campaigns [8]. It was noted that the normalisation of conversations around organ donation was one of the positive factors in the Spanish opt-out system, contributing to the country having improved organ donation rates from 14 to 47 per million population in 2017 [8]. Other studies [10–13] have explored professionals’ knowledge and attitudes towards organ donation and found a lack of knowledge, with professionals feeling uniformed and having varying levels of support and knowledge around organ donation law. Supporting this a review in Wales, carried out 2 years after the introduction of opt-out suggested NHS staff would benefit from further training [14]. This is particularly pertinent as a Welsh Government funded analysis of the influence that media coverage had on public attitudes in the run up to the their legislative change, identified health care professionals as credible sources of information [15]. There are also additional views [16–18] which suggest that the change in legislation is flawed and will increase the number of opt-outs and reduce donation levels. With the above in mind, NHSBT planned their awareness campaign and tracked changes in public awareness and action taken after hearing of the change in legislation [19]. These data show an increase in awareness and positive action taken after hearing of the change in legislation. However, there is still a lack of awareness in ethnic minority groups and at the end of January 2021 ethnic minorities made up 64% of those who had registered an opt-out [19, 20].

This study set out to utilise an online survey named #options, to explore the views of NHS staff in two geographical regions in England. It investigated levels of awareness, support and action taken towards the new organ donation legislation. To better understand what influences opinions, data was collected on sex, age, ethnicity, religion, area and type of work. The findings from this study will deepen the understanding of awareness and support and aid the development of educational resources around organ donation and the change in legislation for NHS staff.

## Methods

An online survey based on the questions used in the Welsh opt-out public survey and NHSBT Kantar population survey was developed and peer reviewed by the NHSBT Implementation team. A copy of the survey is shown in additional information 1. Use of the survey as a clinical research study was approved through the integrated research application system (IRAS) and registered as a National Institute of Health Research (NIHR) portfolio trial [IRAS 275,992]. All NHS organisations in North Thames and the North East and North Cumbria were invited to participate in the study via a feasibility survey sent through the respective NIHR Local Clinical Research Networks in December 2019. These local networks coordinate and support the delivery of research taking place within the NHS in England. Due to the COVID-19 pandemic, the start date was delayed from March to July 2020. Subsequently because of prioritising urgent public health studies, 13 of the 20 acute secondary care Trusts in North Thames who had planned to participate withdrew. Participating organisations (including primary care, secondary care, mental health, ambulance and community services) were given a communications package to advertise the survey via organisational newsletters, direct email and/or internal advertising in staff areas. A list of invited and participating organisations can be found in additional information 2.

The survey collected demographic information on respondents (age, sex, ethnicity, religion), data relating to the NHS organisation they were employed by and level of patient contact (including donor patients and transplant recipients). These factors were used as independent variables in the analysis of the results, for organisation results from the transplanting centre were used as the numerator. Awareness of the change in legislation was measured prior to presenting a short paragraph describing the change in organ donation legislation. The second section of the survey focussed on questions specific to the change in legislation. This included: reflection on own views towards the change, response towards and proposed action to the change in legislation and discussion of their action and decision with family members. Some answers within this section provided a free text space to allow respondents the opportunity to elaborate. Data from these will be reported separately.

### Comparison population data

The data from the #options NHS staff survey was compared against specific matching questions from NHSBT commissioned Kantar population surveys. These surveys are completed monthly in England by individuals over the age of 16 years with sample sizes ranging between 2000 and 2300. For comparison, the Kantar surveys from August to December 2020 were used for analysis. Full data from these surveys is held by NHSBT, reproduced and published with permission from NHSBT and Kantar.

### Statistical analysis

All statistical analyses were performed with Statistical Analysis Systems (SAS) Enterprise Guide v7.1 software (SAS Institute Inc., Cary, North Carolina, United States of America). Differences in categorical variables for demographic data were compared using Chi-squared tests. Multivariable analysis using logistic regression was performed to identify the most significant factors associated with participants’ awareness of and support for the change in legislation and the action following this change (limited to opt-in and opt-out). Participants who were ‘unsure’ as to their opinions of the legislation change were grouped together with participants requiring ‘more information’. A p-value of < 0.05 indicates a statistically significant difference between groups. Data was collected from 5799 members of staff, of which 5789 confirmed that they were happy to complete the survey.

## Results

The questionnaire was completed by 5789 members of NHS staff between 23 and 2020 and 31 December 2020. The majority of respondents were from the North East and North Cumbria (n = 4986, 86%) with a lower response rate from North Thames (n = 803, 14%). A breakdown of the characteristics of the 5789 participants is provided in additional information 3.

### Awareness

Across the study period 68% of NHS staff participants said they were aware of the changes in organ donation legislation, 19% were unaware and 13% were not sure.

The results of the logistic regression for awareness identified the following factors were associated with an increased likelihood of being aware of the change in legislation: being White, being female, working in a transplanting centre, working in an area supporting or providing face to face care for donors and recipients and having discussed organ donation decision with family. The factors associated with being least aware of the change in legislation were: no discussion about organ donation choices with family, working in mental health trust, being Asian (note small number of responses from other ethnic groups) and being male. The odds ratio and p value for factors affecting awareness of the change in legislation are shown in Table 1. Multivariable analysis indicated age (p = 0.38) and religion (p = 0.08) were non-significant.

**Table 1:** Results from multivariable analysis showing impact of demographic factors on awareness of the change to organ donation legislation in England amongst NHS staff September-December 2020

Factor	Number	Odds ratio	95% CI	P value
**Ethnicity (p = 0.001)**				
White	5281	1.0	-	
Asian	212	0.6	0.4–0.8	0.0003
Black	92	1.3	0.8–2.1	0.21
Chinese	29	1.3	0.6–2.8	0.54
Mixed	41	1.9	0.8–4.2	0.13
Other	58	0.6	0.4–1.1	0.10
Prefer not to say	76	0.7	0.4–1.2	0.17
**Age** -multivariable analysis indicated age of responder did not significantly impact on awareness when other variables were accounted for				p = 0.38
**Gender (p = 0.03)**	**Number**	**Odds ratio**	**95% CI**	**P value**
Male	1235	1.0	-	
Female	4506	1.2	1.0–1.4	0.02
Prefer not to say	48	1.6	0.8–3.4	0.18
**Religion** -multivariable analysis indicated religion of responder did not significantly impact on awareness when other variables were accounted for				P = 0.08
**Organisation (p < 0.0001)**	**Number**	**Odds ratio**	**95% CI**	**P value**
Transplanting centre	1022	1.0	-	
Ambulance Service	598	0.6	0.5–0.8	0.0002
Primary Care Services	684	0.5	0.4–0.6	< 0.0001
Other Acute Medical Trust	2713	0.4	0.4–0.5	< 0.0001
Mental Health Trust	687	0.3	0.2–0.4	< 0.0001
Other/Missing	85	0.3	0.2–0.6	< 0.0001
**Work in area supporting donors or recipients (p = 0.0002)**	**Number**	**Odds ratio**	**95% CI**	**P value**
Yes	1560	1.0	-	
No	4162	0.7	0.6–0.8	< 0.0001
Missing	67	0.8	0.4–1.3	0.34
**Face to face contact with donors and recipients (p = 0.01)**	**Number**	**Odds ratio**	**95% CI**	**P value**
Yes	1660	1.0	-	
No	4096	0.8	0.7–0.9	0.004
Missing	33	0.9	0.4–2.0	0.83
**Have you discussed decision with family member (p < 0.0001)**	**Number**	**Odds ratio**	**95% CI**	**P value**
Yes	4359	1.0	-	
No	1430	0.4	0.3–0.4	< 0.0001

Comparison with the NHSBT Kantar survey between September and December 2020 showed that awareness in NHS staff was greater than the general population (68% vs. 60%, p < 0.0001) and that NHS staff from minority ethnic groups had a higher level of awareness than minority ethnic groups from the public survey (57% vs. 45%,  p < 0.0001). In addition, awareness across certain faith groups was higher than comparable data from the NHSBT Kantar survey (p < 0.0001). For example, Muslim: 55% vs. 45%, Buddhist: 70% vs. 35%, Sikh: 73% vs. 42% and Hindu: 57% vs. 38%.

### Support

**Fig. 1:**
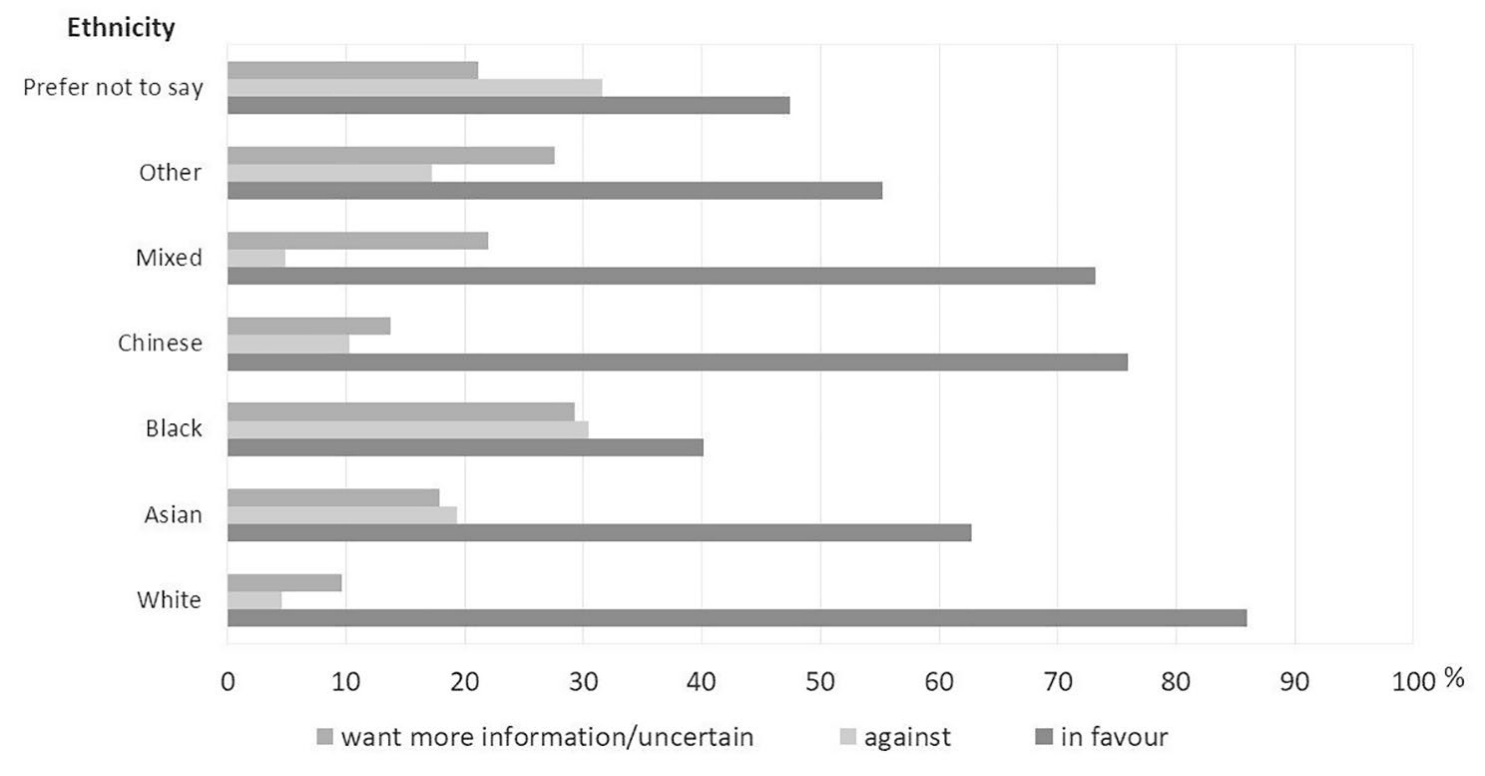
Support of NHS staff to the change in organ donation legislation by Ethnicity, September-December 2020

Overall, 83% of the NHS staff participants were supportive of the change in legislation, 6% were against, 6% needed more information and 5% were unsure. Figure 1 illustrates the impact of ethnicity with lower levels of support in Black and Asian staff when compared to White staff. There were also higher levels for more information required and uncertainty in all ethnic groups when compared to the White ethnicity. Geography also has an association, with higher support in the North East and Cumbria (84%) than North Thames (75%, p < 0.0001).

The results of the logistic regression identified the following factors were associated with lower levels of support for the change in legislation: Black or Asian ethnicity, identifying as Christian, Muslim or Jewish, not discussed organ donation decision with family, older age (> 45 years) and not being aware of the change in legislation until completing the survey. Of the named religions Hinduism was least likely to be associated with lack of support. The odds ratio and p value for factors affecting opinions against that change to organ donation legislation are shown in Table 2.

**Table 2:** Result from multivariable analysis showing impact of demographic factors on opinions against the change to ‘opt-out’ organ donation legislation in England amongst NHS staff September-December 2020

Factor	Number	Odds ratio*	95% CI	P value
**Ethnicity (p < 0.0001)**				
White	5281	1.0	-	
Asian	212	3.5	2.3–5.5	< 0.0001
Black	92	7.2	4.5–11.5	< 0.0001
Chinese	29	1.5	0.6–3.7	0.40
Mixed	41	2.6	1.2–5.5	0.02
Other	58	3.2	1.8–5.9	0.0001
Prefer not to say	76	3.6	2.0–6.4	< 0.0001
**Age group (p < 0.0001)**	**Number**	Odds ratio*	**95% CI**	**P value**
18–24	359	1.0	-	
25–34	1234	1.0	0.7–1.5	0.99
35–44	1279	1.5	1.1–2.2	0.03
45–54	1618	1.8	1.3–2.7	0.001
55+	1244	2.3	1.6–3.3	< 0.0001
Prefer not to say	55	3.6	1.7–7.6	0.0006
**Gender** -multivariable analysis indicated gender of responder was not significant when other variables were accounted for				0.29
**Religion (p < 0.0001)**	**Number**	Odds ratio*	**95% CI**	**P value**
No religion	2560	1.0	-	
Christian	2814	1.4	1.2–1.7	< 0.0001
Muslim	75	2.7	1.5–4.9	0.001
Buddhist	30	0.8	0.3–2.3	0.65
Jewish	29	2.6	1.1–6.1	0.03
Hindu	70	0.3	0.1–0.7	0.003
Sikh	15	0.5	0.1–2.2	0.38
Prefer not to say/other	196	1.9	1.3–2.8	0.002
**Organisation (p = 0.008)**	**Number**	Odds ratio*	**95% CI**	**P value**
Transplanting centre	1022	1.0	-	
Other Acute Medical Trust	2713	0.7	0.6–0.9	0.004
Ambulance Service	598	0.5	0.4–0.8	0.0003
Mental Health Trust	687	0.8	0.6–1.0	0.06
Primary Care Services	684	0.7	0.6–1.0	0.04
Other/Missing	85	1.0	0.5–1.7	0.91
**Working in an area supporting donors or recipients** -multivariable analysis indicated this was not significant when other variables were accounted for.				0.72
**Face to face contact with donors and recipients** -multivariable analysis indicated this was not significant when other variables were accounted for.				0.99
**Have you discussed decision with family member (p < 0.0001)**	**Number**	Odds ratio*	**95% CI**	**P value**
Yes	4359	1.0	-	
No	1430	4.0	3.4–4.7	< 0.0001
**Aware of changes to the organ donation legislation (p < 0.0001)**	**Number**	Odds ratio*	**95% CI**	**P value**
Yes	3950	1.0	-	
No	1073	2.1	1.7–2.5	< 0.0001
Not sure	766	1.8	1.5–2.3	< 0.0001

*Odds Ratio > 1 indicates lower levels of support to ‘opt-out’ legislation, Odds Ratio < 1 indicates higher levels of support to ‘opt-out’ legislation

Support for the change in legislation was high across all NHS workplace organisations with 83% of all respondents indicating support to the change in legislation to ‘opt-out’ but support was highest in those working in the ambulance service (OR 0.5, CI 0.4–0.8, p = 0.0003) with 90% of the respondents supportive. Interestingly univariate analysis showed a larger percentage of participants working in a transplanting centre were against the change when compared to participants working outside of transplanting centres (9% vs 5%) and there were lower levels of uncertainty (8% vs 11%).

The following factors were non-significant when taking account of all factors in the multivariable analysis; gender (p = 0.29), face to face contact with donors and recipients (p = 0.99) and working in an area supporting donors or recipients (p = 0.72).

### Action

**Fig. 2:**
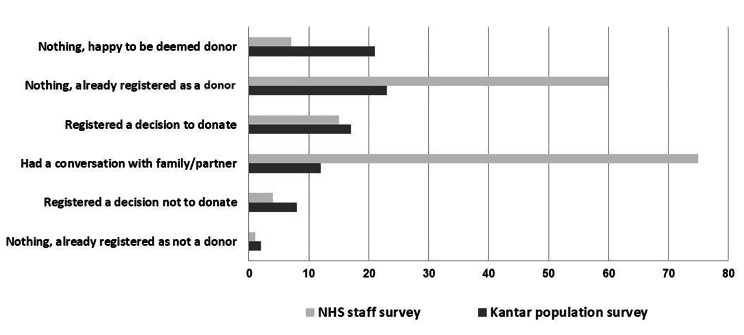
Actions taken by NHS staff in England regarding organ donation, September-December 2020

The action taken by respondents in both the #options survey and NHSBT Kantar population survey is shown in Fig. 2. When compared against the public population univariate analysis showed that NHS staff respondents were much more likely to be on the organ donation register (60% vs. 23%, p < 0.0001). They were more likely to have had a conversation with partner/family (75% vs. 12%) and less likely to have registered a decision not to donate (4% vs. 8%, p < 0.0001). Of note is that respondents may have been referring to some actions and conversations about organ donation that had potentially occurred prior to the change in legislation. Univariate analysis revealed White respondents were more likely to have discussed their decision with a family member than non-White respondents (77% vs. 56%, p < 0.001). But more than half of NHS staff respondents for all minority ethnic groups had spoken to their family about their decision (56%) and 37.5% were on the ORD.

The data across actions and support indicates that a larger percentage of NHS staff participants who had discussed their decision with a family member were in favour of the change when compared with NHS participants who were not supportive or uncertain (89%, 61% and 65% respectively, p < 0.0001).

The survey included a question about planned action in response to the change in legislation; the following responses were included for logistic regression analysis ‘I will register a wish to be a donor’ (opt-in, n = 883) and ‘I will register a wish not to be a donor’ (opt-out, n = 250). The analysis identified that the following factors were associated with planned action to ‘opt-in’ following the change to the legislation: being White, being younger (< 45 years of age), female, of no religion and working in the ambulance service or an acute medical trust. Factors associated with being the least likely to ‘opt-in’ were being Black, Muslim and male. The odds ratio and p value for factors affecting planned action to support ‘opt-in’ following the change in organ donation legislation are shown in Table 3.

**Table 3:** Results from multivariable analysis showing impact of demographic factors on action supporting ‘opt-in’ following the change to organ donation legislation in England amongst NHS staff September-December 2020

Factor	Number	Odds ratio	95% CI	P
**Ethnicity (p < 0.0001)**				
White	993	1.0	-	
Asian	61	0.5	0.2–0.4	0.19
Black	30	0.1	0.05–0.3	< 0.0001
Chinese	10	0.6	0.1–2.4	0.45
Mixed	10	0.4	0.1–1.8	0.23
Other	13	0.2	0.1–0.7	0.01
Prefer not to say	16	0.4	0.1–1.6	0.18
**Age group (p = 0.001)**	**Number**	**Odds ratio**	**95% CI**	**P**
18–24	76	1.0	-	
25–34	194	0.6	0.3–1.4	0.26
35–44	232	0.9	0.4–2.1	0.86
45–54	325	0.4	0.2–0.8	0.02
55+	291	0.5	0.2–1.0	0.04
Prefer not to say	15	0.2	0.04–0.7	0.01
**Gender (p = 0.0003)**	**Number**	**Odds ratio**	**95% CI**	**P**
Male	268	1.0	-	
Female	855	2.0	1.4–2.9	0.0002
Prefer not to say	10	0.3	0.1–2.2	0.26
**Religion (p < 0.0001)**	**Number**	**Odds ratio**	**95% CI**	**P**
No religion	471	1.0	-	
Christian	561	0.7	0.5–1.0	0.07
Muslim	28	0.1	0.0–0.4	0.0004
Hindu	25	5.8	0.9–35.8	0.06
Prefer not to say/other^1^	48	0.4	0.2–0.8	0.01
**Organisation (p < 0.0001)**	**Number**	**Odds ratio**	**95% CI**	**P**
Transplanting centre	144	1.0	-	
Other Acute Medical Trust	574	3.0	1.9–4.6	< 0.0001
Ambulance Service	111	5.2	2.6–10.7	< 0.0001
Mental Health Trust	150	1.9	1.1–3.3	0.02
Primary Care Services	142	2.0	1.1–3.4	0.02
Other/Missing	12	3.7	0.7–19.0	0.12
**Working in an area supporting donors or recipients** -multivariable analysis indicated this was not significant when other variables were accounted for				0.96
**Face to face contact with donors and recipients** -multivariable analysis indicated this was not significant when other variables were accounted for				0.34

The following factors were non-significant when taking account of all factors in the multivariable analysis; face to face contact with donors and recipients (p = 0.96) and work in an area supporting donors or recipients (p = 0.34).

## Discussion

This study contributes to ongoing NHSBT work to track public opinion towards organ donation and provides greater understanding of the awareness and opinions of healthcare workers to the change in legislation. The heightened awareness of NHS staff respondents to the change in organ donation legislation identified through this study is reassuring and positively reflects the impact of the extensive campaign run by NHSBT prior to the change in legislation. This is particularly so, as the #options study collected data during the 2nd wave of the COVID-19 pandemic, a time of significant challenge and learning for NHS staff. As expected, staff working in a transplanting centre were more aware than those working in any other NHS working environment. These findings are similar to those of Young et al. [14] in Wales where higher levels of awareness and knowledge were reported in accident and emergency and intensive care staff. Awareness levels were lowest in respondents working in mental health trusts, being similar to public awareness. Concurring with the findings from Wales, this supports the view that further campaigns to improve awareness and increase knowledge should be engaged with.

Although NHS staff respondents reflect the known public discourse, it is of note that NHS staff respondents were much more aware than the public across two important demographics, those from minority ethnic and religious groups. This offers significant opportunity for NHS staff to both lead and support conversations within their own communities improving awareness about organ donation. Supporting this conclusion, recent work [21] reported an increase in the numbers of those considering organ donation from a religious minority, following a training session delivered jointly by local healthcare professionals and religious leaders.

The level of support for the change in legislation observed in NHS staff respondents in this study is also reassuring, they are higher than levels of support identified in other international studies where there is opt-out legislation [13, 22]. It is comparable to Scotland where prior to the planned change to opt-out it was reported that staff felt it would have a positive impact [23]. Additionally, the Welsh NHS staff survey reported increasing support for the change in legislation from 71% prior to the introduction to 89% two years post introduction in 2017 [24]. #options showed that support among English NHS staff within the first 6 months of the legislation was already 83%. It further indicated that support was positively associated with those who had discussed their decision with a family member. Reflecting the known barriers to donation [2], support for a move to opt-out was lower in NHS staff respondents from the majority of minority ethnic and religious groups. This reflects Etheredge [25] who reports that opt-out systems do not negate as expected the issues of religious and sociocultural preclusion. Innate mistrust of healthcare systems and views about personal choice remain barriers and may be exacerbated by the change in legislation. However, it is of note that 59% of NHS staff respondents from minority ethnic groups still supported the change in legislation as did 45% of Muslim staff respondents. Although there is no direct contemporaneous comparison question in the NHSBT Kantar public survey, this level of support is higher than anticipated from other published data [4, 18]. In addition, a larger proportion of staff from minority ethnic groups were uncertain and/or wanted more information to help them decide, thus offering significant future opportunities to increase awareness and support through education. This view is supported by Vincent et al. [26] who showed that higher knowledge towards organ donation was linked to positive actions among Indians living globally. They also suggested that there is still a considerable sociocultural element irrespective of country of residence with some suggestion that there are differing views across younger and older individuals. The younger generation were more willing to discuss their views with their family, which was significant when consent was requested.

The high levels of support and opt-in actions seen in staff working in the ambulance service is an interesting finding. The cause is not clear and worthy of further investigation. It may reflect the nature of dealing with cardiac arrests and catastrophic emergencies where donation situations may occur. It could be that these staff felt it more appropriate to take the definite action to opt-in rather than be deemed a donor by absence of opt-out. It may also reflect the previously discussed higher levels of knowledge in accident and emergency and intensive care staff found in Wales [14]. For those working in a transplanting centre, there was less uncertainty and the highest level of staff respondents who were against the change. Prior to the change in legislation, NHSBT commissioned a clinical working group exploring the potential impact of any change in legislation [5]. The working group recognised concern from health professionals about the possible negative implications for clinical practice, especially the potential to damage the vital relationship of trust between clinicians caring for people at the end of life, their patients and their families. It was felt this would make the critical care environment more difficult to work in. This concurs with the findings from Brazil where opt-out was reversed after healthcare professionals refused to adopt the new legislation due to its use of a ‘hard opt-out model’ where consent was not sought from relatives [25]. This unease with the potential consequences of the law change was also seen in the data from Wales [14] where it was suggested further training for healthcare professionals was required around their conversations with families at the time of possible donation. It is also suggested in work from Canada where a substantial number of physicians working in a critical care environment expressed neutral or negative opinions around opt-out or mandatory referral legislation [13]. It could also be suggested that the increased likelihood of registering on the ODR of those working in a transplanting centre and the high levels of opt-in from the ambulance staff is a way of aiding colleagues at a time where challenging discussions and decision are being made. This again supports the need for further education and training to assist those working in areas where donation conversations take place. The qualitative analysis of the free text responses will add value by exploring these opinions. However, these findings also support continued review of opinions and actions with additional work exploring the reasons for actions being taken. It is well reported [27–29] that the way information about organ donation is conveyed to relatives has an impact on the decision made. The knowledge and attitudes of healthcare professionals, including the language used, the timing and the compassion conveyed, can have both positive and negative influence.

The association of geography on awareness, support and action with higher levels in the North East and North Cumbria than North Thames is also interesting and reflects other known geographical differences in consent rates and awareness [30].

The field of organ donation and the move to opt-out systems is complex and contradictory. Recent works [9] have suggested that opt-out does not increase the number of donors, where others [31] report the opposite. Opt-out countries reported as successes often have multiple other polices and initiatives aimed at supporting and enhancing organ donation [25]. Opt-out legislation alone will not solve the shortage of organs for donation; the impact of the change in legislation, both positive and negative, requires close monitoring. Ongoing work is required to track both NHS staff and public opinions and suggest further strategies to enhance organ donation rates.

The potential effect of the pandemic on the sample size and make-up of the respondents should not be discounted. During the second wave of the pandemic the Department of Health and Social Care suspended research that was not defined as urgent public health, this included the #options survey. In the North East and Cumbria region the second wave and suspension occurred after the launch of the #options survey. This may have contributed to the disparity in responses between the geographical areas, which in turn affected the ethnic diversity of respondents. However, as there is no previous comparable work in this field it is difficult to assess the degree of impact. Respondents to #options had a similar age [32] and sex [33] profile to the NHS workforce but levels of ethnic diversity were lower [34]. This difference reflects the high response rate from the North East and North Cumbria, where the most recent UK census data showed 93% of the general population are White [35]. This is a limitation to this study. However, it is partially offset by the large number of responses allowing subgroup analysis. A further limitation is the possible effect the COVID-19 pandemic had on staff awareness of current issues. The implementation of the law in May 2020 coincided with the first wave of the pandemic a time when all healthcare organisations were operating under very abnormal systems and processes This may have affected any planned increase in education and training and therefore awareness. However, these findings shed useful light on the situation at the time and form part of ongoing monitoring.

## Conclusion

NHS staff respondents from this study demonstrated they were well informed about the change in legislation. When comparing NHS staff against the public the NHS staff were more aware, supportive and more likely to have taken positive action around the change in organ donation legislation, even across religious and minority ethnic groups. The findings of this study show a consistent strong relationship between NHS staff respondent awareness and support for the change in legislation, this is replicated across all age groups, men and women, ethnicity and religion. The factors influencing these interactions are complex but support the critical role of education and publicity campaigns within the NHS and wider public to impact in a timely and positive way upon individual support and action. The higher levels of uncertainty and requests for further information from minority ethnic groups suggest that educational packages could be tailored to and delivered to specific groups.

One of the most significant findings of this study is the high level of positive action taken in response to the change in legislation and the number of staff who have had conversations with their family and friends. This opens multiple new opportunities to support NHS staff, where appropriate, to be advocates and ambassadors for organ donation and the change in legislation, for example within primary care and community groups. Further work that explores the views of NHS staff is recommended. This should consider the period of time post enactment of law in England and the knowledge that all four United Kingdom nations now have opt-in legislation.

## Electronic supplementary material

Below is the link to the electronic supplementary material.

**Additional file 1:** #options survey

**Additional file 2:** List of eligible participating organisations

**Additional file 3:** Characteristics of participants

## Data Availability

The datasets analysed during the current study are available from the corresponding author on reasonable request.

## Abbreviations

UK: United Kingdom
NHS: National Health Service
NIHR: National Institute for Health and Care Research
ODR: Organ Donation Register
NHSBT: National Health Service Blood and Transplant
IRAS: Integrated Research Application System
COVID-19: Coronavirus Disease 2019
SAS: Statistical Analysis System
