# Supplementary material for: NHS staff awareness, attitudes and actions towards the change in organ donation law in England—results of the #options survey 2020
Source: Arch Public Health. 2023 May 10;81:88. doi: 10.1186/s13690-023-01099-y (PMC10170439; doi:10.1186/s13690-023-01099-y)
Supplement: Supplementary file 1 — Additional file 1: #options survey [file 13690_2023_1099_MOESM1_ESM.docx]

**Additional file 1: #options survey**

Q1 I confirm I am happy to complete this survey Yes/No

Q2 Gender male/female/prefer not to say

Q3 Age group 18-24

25-34

35-44

45-54

55+

Q4 Which of the following categories best describes your ethnic group?

White British

White Irish

Any other white background

Asian or Asian British-Pakistani

Asian or Asian British-Indian

Black or Black British-African

Black or Black British-Caribbean

Chinese

Mixed-white and Asian

Any other

Q5 Which of the following categories best describes your religion?

No religion

Christian

Muslim

Buddhist

Jewish

Hindu

Sikh

Prefer not to say

Q6 Which region do you work in?

North Thames

North East and North Cumbria

Q7 What type of organisation do you work in

NHS Primary care organisation

NHS Secondary care organisation

NHS Other including ambulance service

Q8 Which organisation do you work in?

Drop down menu to reflect organisations involved (secondary care organisations only)

Q9 Do you have face to face contact with patients/service users as part of your job?

Y/N

Q10 Do you have face to face contact with transplant recipient or donor patients as part of your job?

Y/N

Q11 Do you work in part of the NHS that provides a service that supports organ donation patients and recipients?

Y/N

Q12 Are you aware of any changes that are taking place to the organ donation system in England from spring 2020?

Yes

No

Not sure

**Please read this statement**

From spring 2020 organ donation in England will move to an ‘opt out’ system. You may have heard it referred to as ‘Max and Keira’s law’. This means that all adults in England will be considered to have agreed to be an organ donor when they die unless they have recorded a decision not to donate or are in one of the excluded groups.

Excluded groups include those under 18 years of age, people who lack the mental capacity to understand, visitors to England and those who have lived in England for less than 12 months before their death.

Q13 Which of these statements about changes to the organ donation system in England best reflect your views as an individual?

1. I am in favour of this change in legislation
2. I am against this change in legislation
3. I need more information to decide
4. I don’t know

Responses will be linked depending upon answer above

1. I am in favour of this change in legislation-move to question 14
2. I am against this change in legislation- can you help us understand why you are against this legislation? (Free text space)
3. I need more information to decide- what information would you like to help you decide? (Free text space)
4. I don’t know -move onto question 14

Q14 When this new system is introduced, which of the following best describes what you think you will do?

- I will register a wish to be a donor (ie opt in)
- I am already on the organ donor register
- I will register a wish not to be a donor (ie opt out)
- I have already opted out on the organ donor register
- I will nominate an individual to make the decision about organ donation on my behalf
- I have already nominated an individual to make the decision about organ donation on my behalf
- I am not on the organ donor register and I do not need to take any action as my consent will be assumed
- I do not know

Q15. Have you discussed your decision with a family member?

Yes

No

If no link to free text box and the following question ‘can you help us understand what has stopped you discussing this with our family?

Q16. Would you be willing to be part of a focus group to help us understand why some people are uneasy about the change in legislation?

YES (if yes link for the person to put their email address in)

No

Thank you for completing the survey, if you would like more information about organ donation or how to opt in or out please click this link

<https://www.organdonation.nhs.uk/>
