# Supplementary material for: NHS staff awareness, attitudes and actions towards the change in organ donation law in England—results of the #options survey 2020
Source: Arch Public Health. 2023 May 10;81:88. doi: 10.1186/s13690-023-01099-y (PMC10170439; doi:10.1186/s13690-023-01099-y)
Supplement: Supplementary file 2 — Additional file 2: List of eligible participating organisations [file 13690_2023_1099_MOESM2_ESM.docx]

**Additional File 2**

**List of eligible participating organisations**

All eligible organisations were invited to participate in the study. Prior to the COVID pandemic 80% of organisations had expressed an interest to participate.

This data contains a list of organisations that participated during the pandemic, showing fewer organisations participated in North West Thames than planned due to the pandemic.

**North East and North Cumbria**

| **Organisation name-Secondary Care** | **Type of Trust** | **Participated?** |
| --- | --- | --- |
| Cumbria, Northumberland Tyne & Wear NHS Foundation Trust | Mental Health | Y |
| Tees, Esk and Wear Valley NHS Foundation Trust | Mental Health | Y |
| County Durham and Darlington NHS Foundation Trust | Acute Medical | Y |
| South Tyneside and Sunderland NHS Foundation Trust | Acute Medical | Y |
| South Tees Hospitals NHS Foundation Trust | Acute Medical | Y |
| Gateshead Health NHS Foundation Trust | Acute Medical | Y |
| Northumbria Healthcare NHS Foundation Trust | Acute Medical | Y |
| Newcastle Upon Tyne Hospitals NHS Foundation Trust | Acute Medical | Y |
| North Tees and Hartlepool NHS Foundation Trust | Acute Medical | Y |
| North Cumbria Integrated Care NHS Foundation Trust | Acute Medical | Y |
| North East Ambulance Service NHS Trust | Ambulance | Y |
| NHS Darlington CCG* | Primary Care | N |
| NHS Durham Dales, Easington & Sedgefield CCG | Primary Care | Y |
| NHS Hambleton, Richmondshire & Whitby CCG | Primary Care | N |
| NHS Hartlepool & Stockton-on-Tees CCG | Primary Care | Y |
| NHS Newcastle Gateshead CCG | Primary Care | Y |
| NHS North Cumbria CCG | Primary Care | Y |
| NHS North Durham CCG | Primary Care | Y |
| NHS North Tyneside CCG | Primary Care | Y |
| NHS Northumberland CCG | Primary Care | Y |
| NHS South Tees CCG | Primary Care | N |
| NHS South Tyneside CCG | Primary Care | Y |
| NHS Sunderland CCG | Primary Care | N |

*CCG=Clinical commissioning group-an area group of GP practices in England

**North West Thames**

| **Organisation name** | **Type of Trust** | **Participated?** |
| --- | --- | --- |
| Central and North West London NHS Foundation Trust | Mental Health | Y |
| Central London Community Healthcare NHS Trust | Community | Y |
| Chelsea and Westminster Hospitals NHS Foundation Trust | Acute Medical | N |
| Imperial College Healthcare NHS Trust | Acute Medical | N |
| London North East University Healthcare NHS Trust | Acute Medical | Y |
| The Hillingdon Hospital NHS Trust | Acute Medical | N |
| West London NHS Trust | Mental Health | N |
| London Ambulance Service NHS Trust | Ambulance | N |
| NHS Herts Valleys CCG* | Primary Care | N |
| NHS Luton CCG | Primary Care | N |
| NHS Mid Essex CCG | Primary Care | Y |
| NHS Thurrock CCG | Primary Care | Y |
| NHS West Essex CCG | Primary Care | N |
| NHS Barking and Dagenham CCG | Primary Care | Y |
| NHS Barnet CCG | Primary Care | N |
| NHS Camden CCG | Primary Care | N |
| NHS City And Hackney CCG | Primary Care | N |
| NHS Enfield CCG | Primary Care | N |
| NHS Haringey CCG | Primary Care | N |
| NHS Havering CCG | Primary Care | Y |
| NHS Islington CCG | Primary Care | N |
| NHS Newham CCG | Primary Care | N |
| NHS Redbridge CCG | Primary Care | Y |
| NHS Tower Hamlets CCG | Primary Care | N |
| NHS Waltham Forest CCG | Primary Care | Y |
| NHS Basildon and Brentwood CCG | Primary Care | Y |
| NHS Castle Point and Rochford CCG | Primary Care | N |

*CCG=Clinical commissioning group-an area group of GP practices in England
