# Supplementary material for: NHS staff awareness, attitudes and actions towards the change in organ donation law in England—results of the #options survey 2020
Source: Arch Public Health. 2023 May 10;81:88. doi: 10.1186/s13690-023-01099-y (PMC10170439; doi:10.1186/s13690-023-01099-y)
Supplement: Supplementary file 3 — Additional file 3: Characteristics of participants [file 13690_2023_1099_MOESM3_ESM.docx]

| **Additional file 3: Characteristics of participants** |  |  |
| --- | --- | --- |
| Category/level | N | % |
|  |  |  |
| Gender |  |  |
| Male | 1235 | 21.33 |
| Female | 4506 | 77.84 |
| Prefer not to say | 27 | 0.47 |
| Missing | 21 | 0.36 |
|  |  |  |
| Age group |  |  |
| 18-24 | 359 | 6.20 |
| 25-34 | 1234 | 21.32 |
| 35-44 | 1279 | 22.09 |
| 45-54 | 1618 | 27.95 |
| 55+ | 1244 | 21.49 |
| Prefer not to say | 36 | 0.62 |
| Missing | 19 | 0.33 |
|  |  |  |
| Ethnicity |  |  |
| White British | 5014 | 86.61 |
| White Irish | 92 | 1.59 |
| White Other | 175 | 3.02 |
| Asian Indian | 121 | 2.09 |
| Asian Pakistani | 30 | 0.52 |
| Asian Other | 61 | 1.05 |
| Black African | 66 | 1.14 |
| Black Caribbean | 21 | 0.36 |
| Black Other | 5 | 0.09 |
| Chinese | 29 | 0.50 |
| Mixed White and Asian | 33 | 0.57 |
| Mixed Other | 8 | 0.14 |
| Other | 58 | 1.00 |
| Prefer not to say | 52 | 0.90 |
| Missing | 24 | 0.41 |
|  |  |  |
| Religion |  |  |
| No religion | 2560 | 44.22 |
| Christian | 2814 | 48.61 |
| Muslim | 75 | 1.30 |
| Buddhist | 30 | 0.52 |
| Jewish | 29 | 0.50 |
| Hindu | 70 | 1.21 |
| Sikh | 15 | 0.26 |
| Prefer not to say | 116 | 2.00 |
| Missing/Other | 80 | 1.38 |
|  |  |  |
| Region |  |  |
| North East and North Cumbria | 4986 | 86.13 |
| North Thames | 803 | 13.87 |
|  |  |  |
| Organisation |  |  |
| Transplanting centre | 1021 | 17.64 |
| Ambulance Service | 598 | 10.33 |
| Other | 3388 | 58.52 |
| Missing | 782 | 13.51 |

|  |  |  |
| --- | --- | --- |
| Category/level | N | % |
|  |  |  |
| Type of organisation |  |  |
| NHS Primary | 684 | 11.82 |
| NHS Secondary | 4233 | 73.12 |
| NHS Other | 807 | 13.94 |
| Missing | 65 | 1.12 |
|  |  |  |
| Mental health trust |  |  |
| Yes | 363 | 6.27 |
| No | 4044 | 69.86 |
| Missing | 1382 | 23.87 |
|  |  |  |
| Face to face contact with patients/service users |  |  |
| Yes | 4274 | 73.83 |
| No | 1491 | 25.76 |
| Missing | 24 | 0.41 |
|  |  |  |
| Face to face contact with recipients/donors |  |  |
| Yes | 1660 | 28.68 |
| No | 4096 | 70.75 |
| Missing | 33 | 0.57 |
|  |  |  |
| Work in area supporting donors or recipients |  |  |
| Yes | 1560 | 26.95 |
| No | 4162 | 71.89 |
| Missing | 67 | 1.16 |
|  |  |  |
| Are you aware of any changes taking place to the organ donation system in England |  |  |
| Yes | 3950 | 68.23 |
| No | 1073 | 18.54 |
| Not sure | 766 | 13.23 |
|  |  |  |
| Opinions about changes to the organ donation legislation |  |  |
| In favour | 4817 | 83.21 |
| Against | 353 | 6.10 |
| More information | 327 | 5.65 |
| Don't know | 292 | 5.04 |
|  |  |  |
| Action following introduction of new legislation |  |  |
| Register wish (opt-in) | 883 | 15.25 |
| Already on ODR | 3445 | 59.51 |
| Register wish (opt-out) | 250 | 4.32 |
| Already opted-out | 79 | 1.36 |
| Nominate individual | 159 | 2.75 |
| Already nominated individual | 40 | 0.69 |
| Not on ODR (assumed) | 434 | 7.50 |
| Don't know | 499 | 8.62 |
|  |  |  |
| Have you discussed decision with family member |  |  |
| Yes | 4359 | 75.30 |
| No | 1430 | 24.70 |
|  |  |  |
